# Usability Testing of a Web-Based Empathy Training Portal: Mixed Methods Study

**DOI:** 10.2196/41222

**Published:** 2023-04-04

**Authors:** Michelle Lobchuk, Lisa Hoplock, Nicole Harder, Marcia Friesen, Julie Rempel, Prachotan Reddy Bathi

**Affiliations:** 1 College of Nursing Rady Faculty of Health Sciences University of Manitoba Winnipeg, MB Canada; 2 Price Faculty of Engineering University of Manitoba Winnipeg, MB Canada; 3 Department of Computer Science and Engineering Manipal Institute of Technology Manipal India

**Keywords:** web application, usability, mixed design research, internet, empathy, mobile phone, mobile devices

## Abstract

**Background:**

The prepandemic period saw a rise in web-based teaching. However, web-based tools for teaching the essential clinical skill of cognitive empathy (also known as perspective taking) remain limited. More of these tools are needed and require testing for ease of use and understanding by students.

**Objective:**

This study aimed to evaluate the usability of the *In Your Shoes* web-based empathy training portal application for students using quantitative and qualitative methods.

**Methods:**

This 3-phase formative usability study used a mixed methods design. In mid-2021, we conducted a remote observation of student participants interacting with our portal application. Their qualitative reflections were captured, followed by data analysis and iterative design refinements of the application. Overall, 8 third- and fourth-year nursing students from an undergraduate baccalaureate program at a Canadian university, in the western province of Manitoba, were included in this study. Participants in phases 1 and 2 were remotely observed by 3 research personnel while engaged in predefined tasks. In phase 3, two student participants were asked to use the application as they liked in their own environments, after which a video-recorded exit interview with a think-aloud process was conducted as participants responded to the System Usability Scale. We calculated descriptive statistics and performed content analysis to analyze the results.

**Results:**

This small study included 8 students with a range of technology skills. Usability themes were based on participants’ comments on the application’s appearance, content, navigation, and functionality. The biggest issues that participants experienced were with navigating the application’s “tagging” features during video analysis and the length of educational material. We also observed variations in 2 participants’ system usability scores in phase 3. This may be because of their different comfort levels with technology; however, additional research is required. We made iterative refinements to our prototype application (eg, added pop-up messages and provided a narrated video on the application’s “tagging” function) based on participant feedback.

**Conclusions:**

With increasing engagement in web-based teaching, technology has become an essential medium for receiving health care education. We developed a novel prototype application as a supplemental classroom tool to foster students’ self-directed learning of empathy. This study provided direction for refinements to optimize the usability of and satisfaction with this innovative application. Qualitative feedback revealed favorable input toward learning perspective taking place on the web and helpful recommendations for improving user experiences with the application. We could not fully assess the application’s key functions owing to the COVID-19 protocols. Thus, our next step is to obtain feedback from a larger sample of student users, whose experiences performing “live” video capture, annotation, and analysis will be more authentic and wholesome with the refined application. We discuss our findings in relation to research on nursing education, perspective taking, and adaptive e-learning.

## Introduction

### Background

The call to action from Taylor et al [1] underscores the need for nurse educators to practice empathy-based teaching methods that foster students’ empathic stance during these unprecedented pandemic times. Without a specific direction in their basic education, students struggle to respond well [2,3]. Thus, to remain resilient in today’s care environment, students and new graduates need to be equipped with self-awareness and emotional self-regulation (the ability to tolerate difficult emotions when confronted with someone else's distress without becoming overwhelmed by those emotions). These skills are salient to the empathic stance [1]. Unfortunately, students, new graduates, and experienced clinicians tend to be prescriptive instead of being empathetic with patient decision-making [4]. Being prescriptive instead of empathetic causes patients to feel unsupported and nonautonomous in the process. Therefore, students require innovative empathy-related strategies to learn self-awareness and emotion regulation not only for practicing empathy but also for self-care to prevent burnout in taxing health care environments [1].

Students often ask, “Can empathy be taught?” Fortunately, reviews indicate that yes, empathy is a skill that is responsive to educational interventions [5,6]. The positive impact of empathy interventions on health care providers was demonstrated by 3 meta-analyses of randomized controlled trials [7-9]. Traditional empathy training in nursing education consists of lectures, workshops, and courses [10]. It can include patient narratives and interviews, creative arts, writing and communication skill training [11], and interviews with a simulated patient followed by clinician or simulated patient feedback [12]. However, these methods are restricted in terms of the number of people who can participate at a time and can be costly. With advancements and extensive adoption of web applications and mobile devices (eg, smartphones, laptops, and tablets), web-based educational methods are supplemental avenues to explore.

### Web Applications and Mobile Devices: Learning Empathy

Before the pandemic, the Canadian Digital Learning and Research Association found growth in web-based learning across universities and colleges [13]. Web-based learning platforms have several benefits for students and institutions, such as cost-effectiveness, flexibility, accessibility, engaging, and self-paced characteristics [14]. Since the COVID-19 pandemic, web-based learning has become more centric, allowing the leveraging of students’ motivation toward and confidence in using technology to practice empathy. Web-based course interventions have a sustained positive influence on empathy; for example, over 2 months of intervention in psychology students had a medium effect size improvement of Hedges *g*=0.66 [15] and over 10 weeks of internship in physical therapy students had a large effect size improvement of Cohen d=1.14 [16]. Mobile devices have also been used to provide instructor feedback in nursing skill training. For example, a Chinese smartphone study examined the use of WeChat (Tencent Holdings Ltd) and QQ (Tencent Holdings Ltd) by nursing students to receive instant instructor feedback on their recorded nursing skill performance. Student comments supported the incorporation of video feedback in mobile technology to improve nursing skills in an economical and accessible manner [17]. Thus, there is some evidence of the benefits and acceptance of using mobile technology in higher education.

Interestingly, in this study, some web applications offered targeted exercises to foster the cognitive empathic process of *perspective taking* [18]. Perspective taking plays an essential role in the clinical understanding of patients [19]. Perspective taking is the imaginative ability to step inside someone else’s shoes to understand their thoughts and feelings, validate the understanding, and act on the understanding in a helpful manner [14]. The range and availability of web applications to cultivate perspective taking are growing in the areas of health, mood states, social connections, games, and social changes [20].

Many perspective-taking apps are geared toward children and youth (eg, *Toca Pet Doctor* [21] and *Peek a Zoo* [22]) and use stories and games. For adult users, a smaller number of web applications focus on promoting the real-life impact of perspective taking. For instance, the ecological momentary assessment intervention provides prompts and exercises for students to integrate empathy-related exercises into daily life. A web-based exercise cultivates accurate perceptual understanding through training in accurate emotion recognition [23]. A commercial product called *In Their Shoes* enables students to live and understand the life of a person with chronic disease through scenarios delivered by smartphone technology and an avatar [24]. Another example is *HeartChat*, a mobile app with a chat feature where physiological data are shared between participants to foster social interaction, connectivity, and awareness. The goal of sharing heart rates is to help people implicitly understand each other’s context (eg, location and physical activity) and emotional state and spark curiosity on special occasions [25]. A final example is the commercially available *Empathy Set* app, which enables participants to identify and track what they and others feel and need through the use of web-based situation-specific flashcards. It also allows them to brainstorm potential solutions and prepare “I” statements so that they can communicate positively [26]. Further advances can be made to existing empathy apps, for example, by providing video feedback on dialogue communication and one’s perspective-taking skills.

Mobile technology enables dialogue partners to record their communication approaches and immediately receive an automatically calculated perceptual understanding score (ie, an “empathic accuracy” score). An empathic accuracy score reflects one’s ability to accurately infer another person’s thoughts and feelings by comparing one’s inferences with another’s self-reports. Empathic accuracy is the outcome of engaging in a perspective-taking process. It has been argued to be a more ecologically valid measure of thought and feeling recognition that requires the real-time integration of visual, auditory, and linguistic information [27]. Empathic accuracy may be a more sensitive measure for the detection of changes in the skills underlying empathy, such as the recognition of thoughts and feelings, that constitute the more cognitive components of empathy. To incorporate the features of video capture and feedback, Lobchuk et al [28,29] created a novel web-based prototype of an empathy-related video feedback intervention called *In Your Shoes* (IYS) to improve perspective-taking skills and empathic accuracy outcomes.

### The IYS Intervention

IYS was adapted from the research paradigm of Ickes et al [30,31]. This paradigm provides a practical, reliable, and objective method for measuring one’s ability to accurately infer another person’s thoughts and feelings during a video-recorded interaction. Quasi- and full-scale intervention studies conducted in the laboratory of Lobchuk et al [28,29] provide evidence to support the work of Ickes et al [30,31] and this study: (1) moderate effect size (Cohen *d*=0.43) difference, where nursing students reported increased empathy in the posttest condition; (2) increased empathic accuracy; and (3) reduced judgment. However, this version of IYS requires attendance in a laboratory setting and a desktop and commercial video analysis software program. To promote accessibility, we transformed our existing in-laboratory desktop method into a unique interactive IYS web application for easy use on any computer or mobile device.

This work-in-progress paper focuses on the development and assessment of a theoretically based educational version of an interactive software application for undergraduate nursing students. The aim of the app is to help nursing students learn how to engage in perspective taking with their patients. Both the desktop and mobile application versions foster perspective taking by providing students the opportunities to practice self-awareness of personal values and emotions that can thwart empathy [28,29], receive instruction [32] and feedback [33], experience empathy [23], and engage in self-evaluation with video feedback [34]. University instructional designers conducted an accessibility check based on World Wide Web Consortium standards and offered pedagogical suggestions, which we addressed in the application. The IYS web application allows students to engage in video capture and annotation and to receive objective empathic accuracy scores to enhance their understanding of patients’ needs, preferences, and beliefs related to care.

### The IYS Application

Full details of the application development process have been reported elsewhere [35]. The IYS web application consists of the following features and tasks for students to learn and practice their perspective-taking skills in the order in which they have been presented in this section:

#### First Step: Landing Page

**Figure 1:**
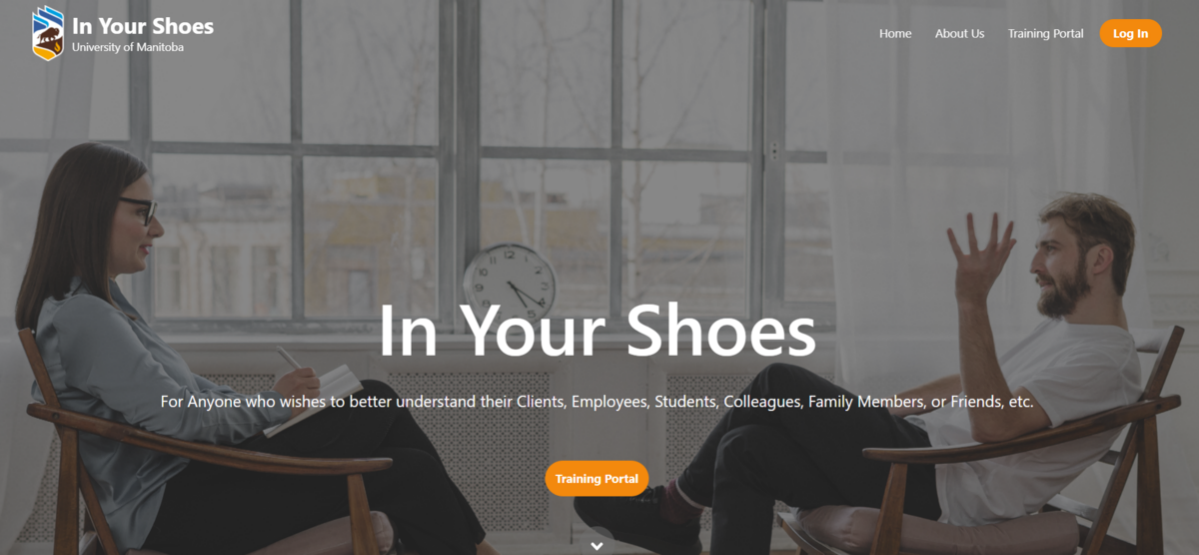
Landing page.

The landing page of the application (Figure 1) consists of the following elements: the target user audience, a link for immediate access to the training portal (no subscription is required during the developmental stage), key app features, pop-up definitions for perspective taking and empathic accuracy scores, steps in learning how to perspective take using the app’s functions, expected outcomes of learning how to perspective take, next steps (after viewing the landing page), and subscription options (for future commercial use).

#### Second Step: Training Portal

**Figure 2:**
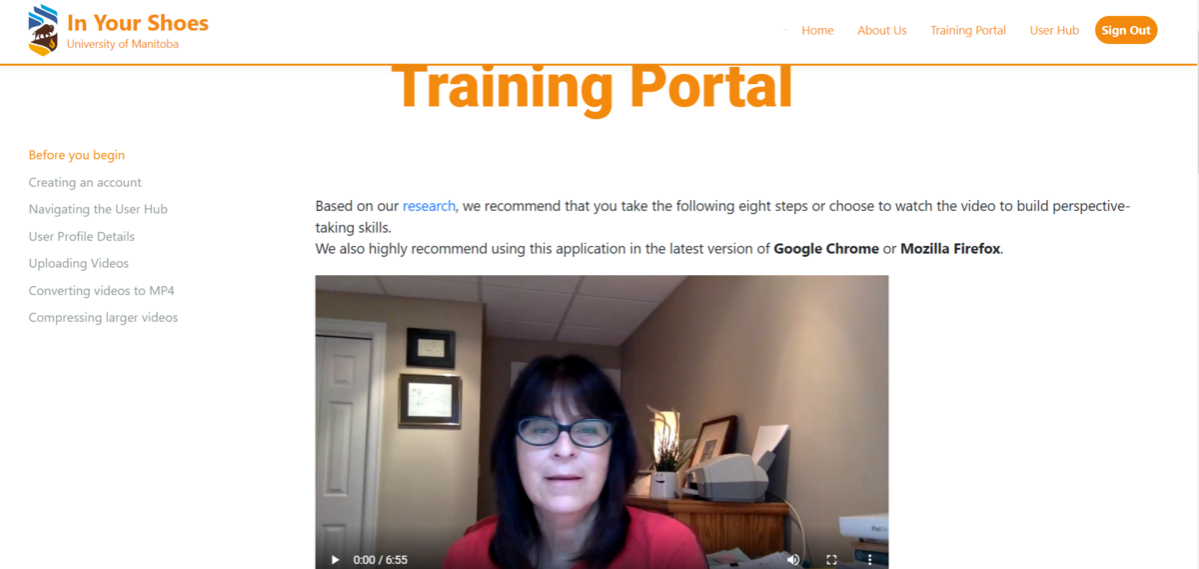
Training portal.

The training portal (Figure 2) consists of a video on perspective taking narrated by the lead author (ML) and 8 stepwise training documents in PDF format. These training documents focus on empathy and its dimensions (with a video by an empathy expert, Dr Daniel Goleman), learning and practicing perspective taking, guidelines for video recording and annotation (“tagging”), and how empathic accuracy scores are calculated by the application’s algorithm.

#### Third Step: The Hub

**Figure 3:**
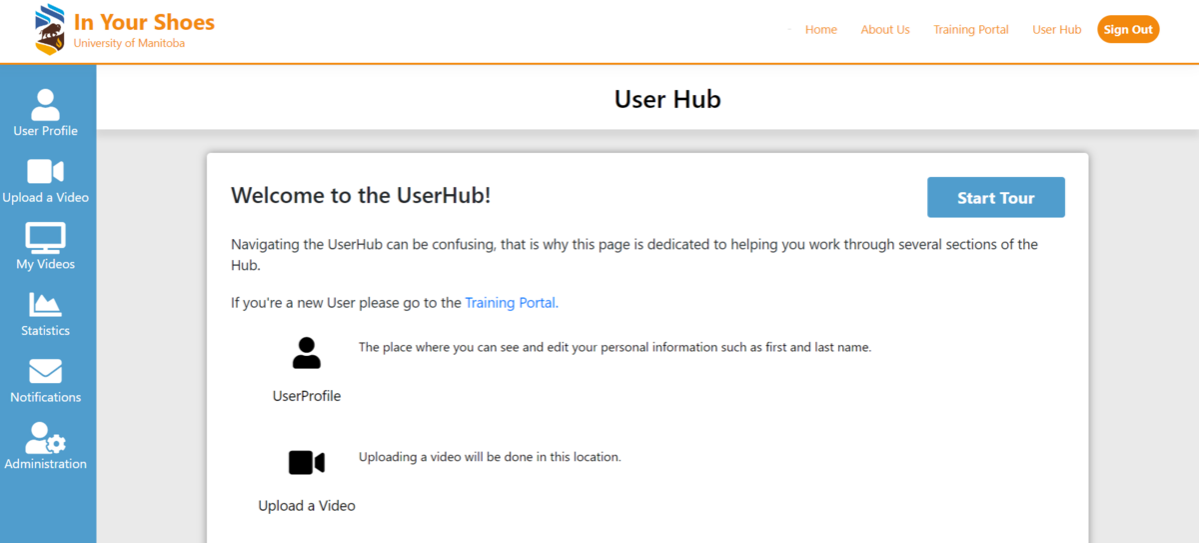
The hub.

The hub page (Figure 3) is where students can view the website’s main features after they have created an account and signed in. By clicking the user hub tab near the top of the screen, student can access the following menu buttons: “user profile,” a link to the section where profile details can be found; “upload a video,” a link to the location for uploading videos; “my video,” a link to the location where videos in various stages of annotation can be found; “statistics,” a link to the section where personal statistics on empathic accuracy scores can be found; “notifications,” a link to the location where notifications and messages are stored; and “administration,” a link for the application’s administrator to view all users, all videos, and all messages.

#### Fourth Step: Video Record and Upload

**Figure 4:**
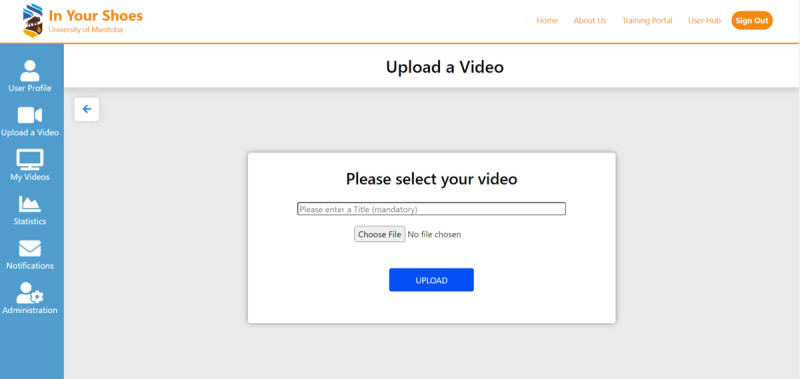
Upload a video recording.

Students identify a conversation topic of importance to them and designate who will play the perceiver and target dialogue partners. The perceiver uses the perspective-taking approach when they dialogue with the target. They video record a “live” 10-minute dialogue in person or on the web to be uploaded to the application for analysis (Figure 4).

#### Fifth Step: Video Annotation

**Figure 5:**
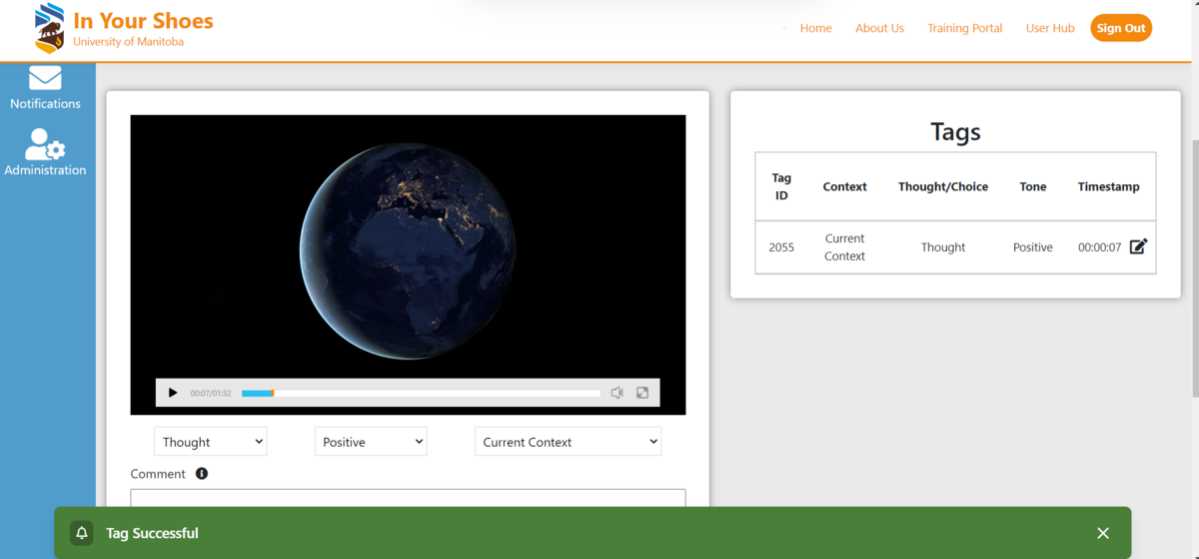
Video annotation.

The target and then the perceiver watch the video recording and report on (or “tag”) each instance where they had a thought or feeling (Figure 5). The application has a drop-down menu of response options that can be used to tag each instance: (1) thought or feeling, (2) tone (neutral, positive, or negative), and (3) situation or context for what the thought or feeling was about (self, dialogue partner, another person or persons, current situation, or another situation).

#### Sixth Step: Obtain Feedback

**Figure 6:**
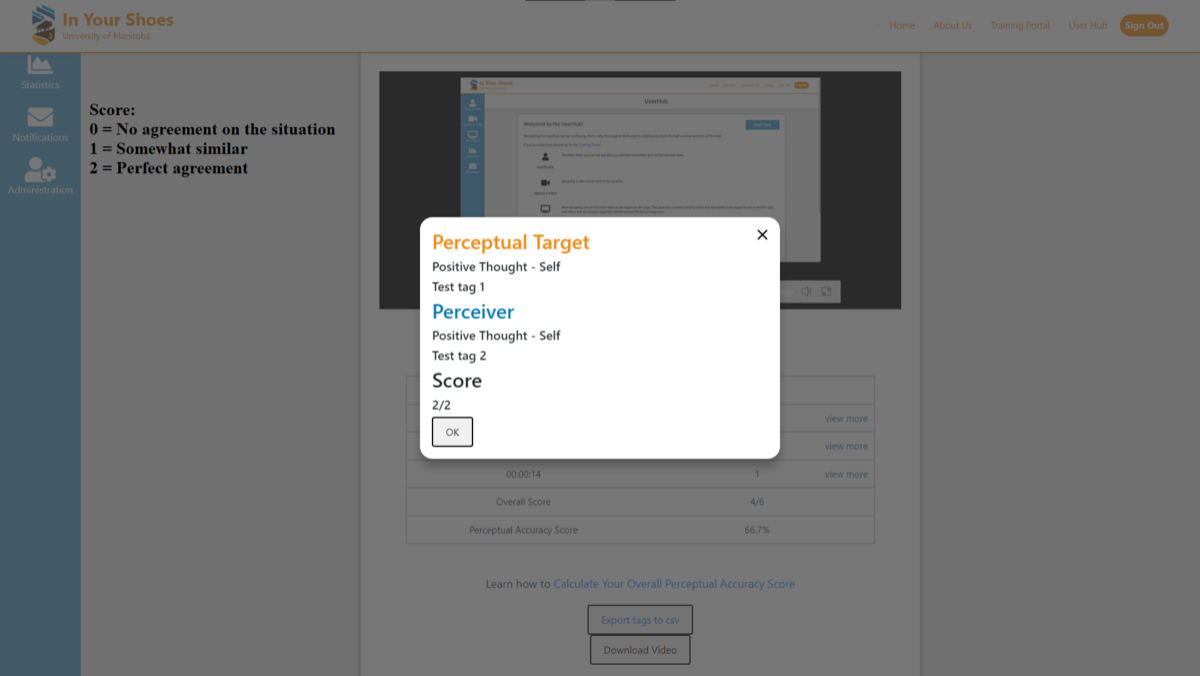
Obtain feedback.

The perceiver and target retrieve and discuss their respective annotated data at each instance captured in the video recording timeline. The perceiver can also access their accuracy score at each instance (Figure 6). The application’s algorithms calculate accuracy scores for each annotated instance. Scores range from “0” to “2” (0=no agreement, 1=somewhat similar, and 2=perfect agreement) across the 3 dimensions. A total percent empathic accuracy score is also calculated by the app.

The IYS web application is unique for several reasons. First, IYS targets higher-education users and adult learners, such as student nurses, and provides flexible discipline-relevant empathy training. Health care educators who desire innovative ways to teach empathy may see this application as a solution if students report that the application offers a compelling learning experience. Another unique feature is that students can practice perspective taking “live” with another person and receive feedback in multiple ways: (1) from the other person or the instructor, (2) by evaluating themselves on video, and (3) from the empathic accuracy score. Just watching a video or looking at a picture and trying to infer what others are thinking and feeling does not lead to perceptual understanding; having a dialogue with others is critical, and seeing oneself on a video is vital for self-reflection and awareness in therapeutic conversations [34]. Furthermore, students can contextualize the dialogue according to their learning needs. Students can also retain and revisit annotated and time-stamped video-captured conversations to monitor improvements in perspective-taking skills over time. This application is intended for prelicensure and licensed clinicians to enhance their perspective-taking skills and empathic understanding. Our next step was to capture students’ intuitive expectations regarding navigating screens and to facilitate engagement with the IYS web application.

### Study Aim and Research Question

Our aim was to evaluate the usability of the IYS web application for teaching perspective taking in a self-directed and engaging manner to nursing students. This study entailed capturing student participants’ preferences and prioritization of features and functionality. The research question was as follows: “How do nursing students perceive and experience the IYS web-based empathy training portal application?”

## Methods

### Study Design

**Figure 7:**
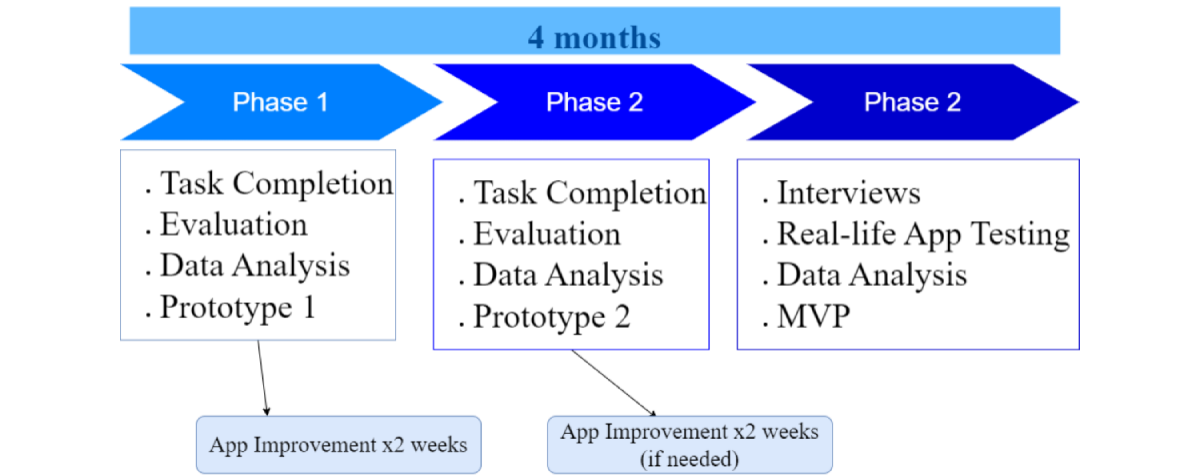
Phases 1 to 3. MVP: minimum viable prototype.

We used a mixed methods design in this 3-phase formative usability study (Figure 7). Phase 1 entailed “live” remote monitoring and analysis of the participants’ video-recorded 1-hour “performance” of requested tasks on the participant’s own device and in the participant’s own environment. This was followed by an analysis of the participants’ feedback and then adjustments to the application.

Phase 2 immediately followed with different participants and involved a “live” remote monitoring and analysis of the participants’ 1-hour video-recorded interactions on the participants’ own device and in the participants’ own environment with the adjusted application [36]. After phase 2, based on the analysis of participant feedback, more adjustments were made to the application. For phases 1 and 2, the trained observers used metrics (described later in the *Data Collection, Phases 1 and 2* section) developed by Tullis and Albert [36].

In phase 3 (“real-life application testing”), different participants independently used the application on their own devices for 1 week without being monitored in their own environment. This was followed by a 30-minute video-recorded, scripted interview that covered participants’ “satisfaction” [36] by focusing on participants’ experiences with, and expectations for, the IYS web application; language understandability; and visual-interactional appeal. Participants also completed a quantitative measure about their experiences with the application. The feedback we received from participants aided in the development of a minimum viable prototype of the IYS application for testing its commercial readiness and reliability for providing training on perspective taking.

### Ethics Approval and Consent

Ethics approval (HS24965; R1-2021-082) and access approval were obtained from the College of Nursing before commencing any study protocols. Before commencing the study, participants were provided with a written study invitation and an informed consent form. Participants were informed that their participation was voluntary and that they could withdraw without prejudice up to 2 days after the exit interview. Participants received a university bookstore gift card worth CAD $50 (US $36.32) at the time of consent. Participants were told that they could keep the gift card even if they decided to withdraw. The consent form explained that the participants' identity would not be revealed under any circumstances. The video recording and closed captions of participants’ responses would not be seen or used by anyone other than the research assistants and lead author for the sole purpose of this project. Pseudonyms (eg, “participant”) were used when direct quotations from the usability test sessions and exit interviews were used to report usability project findings.

### Setting and Sample

This 1-year study took place in mid-2021 at an undergraduate nursing program located in the University of Manitoba. The initial sample size target was 5 participants per phase (3 phases), amounting to a total of 15 participants. This target was based on sampling and recruitment guidelines for usability studies to maximize the expected level of problem discovery [37]. Owing to recruitment and scheduling challenges, the sample comprised 3 third-year undergraduate nursing students and 5 fourth-year students (N=8) who had access to a camera-equipped PC, an Apple desktop computer (Apple Inc), or a tablet device and any browser. Two second-year students and 1 third-year student consented to participate but were difficult to reach to schedule a user-testing session. Each phase comprised the following participants: phase 1 included a fourth-year woman, a third-year man, and a third-year woman (3/8, 38%); phase 2 included a third-year woman, a fourth-year man, and a fourth-year woman (3/8, 38%); and phase 3 included 2 fourth-year women (2/8, 25%). The faculty’s research office coordinator sent an initial email invitation to all undergraduate students (around 800), followed by 2 email reminders at 1-week intervals. Students emailed the research assistant to ask questions about the study. A suitable time was arranged for those who were willing to participate.

### Preparation

Undergraduate students in computer science and computer engineering were hired as research assistants. One of the research assistants was the lead remote moderator, who facilitated the testing sessions using the Microsoft Teams screen-and-audio sharing tool (Microsoft Corp). Two research assistants served as remote silent observers. They watched and recorded the participants’ behaviors during the tasks and made application refinements based on participants’ input. An expert in human-computer interaction trained the research assistants on conducting observations, record keeping in Excel (Microsoft Corp), prompting participants to think aloud while performing tasks, and conducting exit interviews. The remote moderator gave participants task scenarios to perform in realistic steps (eg, setting up an account) [38]. A pilot test was conducted by the remote moderator and remote observers with 2 nursing students. Planning ensured good lines of communication and coordination of “testing” protocols between the research personnel. A detailed description of the role expectations for, and experiences of, the remote moderator and remote observers is provided elsewhere [35].

### Data Collection

Participants differed in each phase. Before engaging in testing protocols, participants completed the informed consent form and a demographic tool in the Qualtrics survey software (Qualtrics International Inc).

#### Phases 1 and 2

The lead remote moderator facilitated the video-recorded interviews and prompted participants to think aloud or talk about anything that they found interesting in the tasks assigned [39]. The remote observers adapted the performance metrics tool developed by Tullis and Albert [36] to capture participants’ responses to 11 predefined tasks (create a new account, log in, upload a video, create a tag, update an existing tag, share a tagged video, export tags in CSV format, download tagged videos, update information, sign out, and reset password). As they observed the test sessions, the remote observers quickly entered numeric indicators on the performance metric tool using Google electronic forms (Google LLC; Multimedia Appendix 1) for the frequency, type, and severity of issues that prevent users from meeting their goals in using the application; task success; common errors; and efficiency (degree of effort and time taken to complete a task). One of the remote observers recorded the participant’s success in completing the predefined tasks, and the second remote observer recorded the number of mouse clicks required to complete each task; this record keeping helped the team ascertain what application refinements were required to improve user experiences.

#### Phase 3

Participants independently used the application on their own devices for 1 week without being monitored. Then, they participated in a 30-minute video-recorded, scripted, and one-to-one exit interview with the remote moderator. The well-known 10-item, 5-point System Usability Scale captured participants’ “satisfaction” based on the perceived effectiveness, efficiency, and overall ease of use of the IYS application [40]. Three added questions captured language understandability, visual-interactional appeal, and whether expectations were met. The System Usability Scale scores are normalized and have a percentile ranking that ranges from 0 to 100; for example, a score of 70 out of 100 suggests a score at the 70th percentile (ie, the application tests above average). A think-aloud process captured participants’ thoughts as they completed the scale. Participants also provided information on the type, brand, and operating system of the device used; number of times they accessed the application; and amount of time they spent on the application each time they accessed it over the past week.

### Data Analysis

Descriptive statistics comprised sample characteristics, performance metrics, and the System Usability Scale scores. ML conducted content analysis and used constant comparison techniques to identify, code, categorize, classify, and label the primary patterns in the transcribed data [41]. On a weekly basis, the research assistants reviewed the transcribed feedback for discussion with ML and to prioritize application refinements before advancing to the next phase.

## Results

### Participant Characteristics

Of the 8 participants, 6 (75%) were women. Participants’ ages ranged from 20 to 35 years. Participants used a range of devices (laptop, smartphone, tablet, and desktop computer), but most (6/8, 75%) used a laptop daily. An Apple device with a Mac operating system was most often used daily (11/19; 58%). All participants reported conducting research, emailing, instant messaging, learning via the web, shopping, paying bills, banking, performing multimedia projects, and interacting with social media on their devices. The least common activities were gaming (3/8, 38%) and engaging with databases (3/8, 38%) and spreadsheets (4/8, 50%). Most participants reported uploading and downloading videos (5/8, 63%) and using a web application daily as part of their web-based education (6/8, 75%). During the testing sessions, all students (8/8, 100%) used Wi-Fi, and most (6/8, 75%) used their laptops, which were most often a MacBook (5/6, 83%; refer to Multimedia Appendix 2 for demographics).

Owing to this study’s small sample size, a caveat regarding the representativeness of the qualitative and quantitative results is warranted. A cursory analysis of gender differences revealed equal proportions of men and women in years 3 and 4 of their study programs. In comparison with the women, the men stated that they seldom or never uploaded or downloaded videos. In addition, one man said that he used a web application for web-based education daily, whereas the other man stated that he “seldom” did. All women stated that they used web applications for web-based education.

### Qualitative Analysis

#### Phases 1 and 2: Overview of Participants’ Responses While Engaging in Tasks

##### Themes

The following subsections on themes include some illustrative quotations that capture participants’ think-aloud responses and feedback from phases 1 and 2. Feedback was classified according to the 4 main areas from Bingham [42]: appearance, content, navigation, and functionality. A summary report on all participants’ feedback and the resulting application refinement action steps are presented in Multimedia Appendix 3. Across phases 1 and 2, participants mainly offered one-off comments within each feedback area.

##### Appearance

The appearance theme captured participants’ feedback on the application’s visual attractiveness, the readability of text (eg, color and font size), engaging features, and the illustrative use of figures and graphics.

This theme was commented on the least. When participants did offer a comment, the issues raised were easily fixed, such as using a larger font size on the application’s landing page and incorporating the use of subheadings to enhance flow. They also recommended inserting attractive features (eg, graphics, animation, or screenshots) in the training portal documents to attract and retain participant interest. However, comments on this theme were generally nuanced and not from the same participants across phases. Thus, it was challenging to evaluate whether improvement in the specific appearance issue (ie, small font size and the need for more engaging features) indeed occurred from phase 1 to phase 2. Some illustrative quotations are as follows:

For me that lettering for ‘anyone who wishes to better understand their clients’ is a little small.

Participant 3, phase 1

Like kind of basic, which can be helpful for some people. And for other people, it can kind of loses [sic] them...some people like to have more of a flow, but otherwise it’s fine. [The participant was referring to the appearance of the training portal documents.]

Participant 5, phase 2

##### Content

The content theme captured feedback that identified participants’ experiences with the information provided in the application. Participants commented on the information’s credibility, usefulness, and logical flow or grouping. They also indicated whether the language used was understandable and clear as well as whether a clear explanation of jargon was provided.

The main content area of interest to participants was the training portal. All participants in phase 1 commented on the extensive and confusing details provided in the PDF training documents. For phase 2, we provided screenshots within the training video and training documents to (1) demonstrate the steps involved in learning perspective taking and how to use the application to test for empathic accuracy and (2) clarify jargon (eg, what is a CSV file). Participants shared positive views on learning about the different dimensions of empathy from the expert’s video. Only one participant indicated that they would not feel the need to use the application again once they have learned how to perspective take. The following are some illustrative quotations for the content theme:

I think the whole document about tagging the video was a bit confusing and maybe some screenshots of someone actually tagging a video would be helpful.

Participant 1, phase 1

...I think this is a really good opening. I feel like the video [empathy video by the expert] is very helpful, very constructive and very informative...I liked it...

Participant 5, phase 2

##### Navigation

The navigation theme captured participants’ experiences with wayfinding and signposting (obvious cues that guide participants to their destination, eg, links and referral pathways). These foster application use efficiency and help users notice key application sections.

Several participants emphasized the need to navigate the application pages in an efficient manner. They recommended clearer instructions on moving from the landing page directly to the training portal, creating video annotations, locating one’s empathic accuracy scores once annotation was completed by both dialogue partners, exporting the CSV file, and messaging the dialogue partner to engage in annotation. Some phase 2 participants appreciated the clear training portal options to view the training video, read the training documents, or do both. Some illustrative quotations for this theme include the following:

OK, so when you say you tagged everything, I think it shouldn’t go back to the user help, but it should just go back to my videos. I don’t know if that’s possible.

Participant 3, phase 1

And I appreciate that on the last one it says “go to step three” at the end of the document. That was helpful. Very good for kind of tracking. [The participant was referring to the training document steps]

Participant 5, phase 2

##### Functionality

The functionality theme captured participants’ feedback on how well the app’s features and functions helped them achieve their goals. This theme included comments about glitches that prevented participants from completing tasks and fully engaging with the application’s activities and features.

There were only a few negative comments on this theme. One of the participants felt strongly about receiving instructions in person or during a one-on-one with an instructor regarding how to use the application and its features. Minor glitches included verification emails going to the junk folder, inefficient access to the PDF training documents during annotation, nonclickable images, and having to click twice to sign out. These issues were easily addressed. The following are some illustrative quotations on this theme:

Yeah...I need to see it being used in person. It needs like an instructor with it or something. [This participant preferred in-person instruction on how to use the application]

Participant 3, phase 1

Yeah, and I’m seeing that these icons are very helpful because they do link back to what you have here, so I think that’s good. The black and white. [The participant is referring to icons on the hub page]

Participant 5, phase 2

#### Phases 1 to 3: Overview of Participants’ Exit Feedback

After the completion of all monitored tasks in phases 1 and 2 and nonmonitored use of the application in phase 3, comments on the most liked and least liked features were provided by participants. These comments were also classified according to the 4 main areas from Bingham et al [42] (Multimedia Appendix 4). Variability and inconsistencies were evident in the most liked and least liked comments. Some of the comments are summarized in the following paragraphs.

The most liked comments are the comments on (1) the “visually appealing” appearance of the application’s pages (participants 7 and 8, phase 3); (2) the use of “good” graphics in the hub, clear description of the application’s purpose and empathy, and clear definitions (participant 5, phase 2; and participant 7, phase 3); and (3) the experience of having good navigation or wayfinding, for example, being able to quickly engage with the application’s key features, take appropriate next steps to learn perspective taking, use the application to practice perspective taking, and obtain their empathic accuracy scores (participant 1, phase 1; participant 6, phase 2; and participants 7 and 8, phase 3).

The least liked comments are comments on (1) the experience of reading “extensive” or “overcomplicated” training documents—one of the recommendations was to have pop-up instructions appear directly on the annotation page as participants do the “tagging” exercise for easy reference (participants 1 and 2, phase 1; participant 4, phase 2; and participant 8, phase 3); (2) the preference for clearer instructions on the landing page for participants to first visit the training portal before accessing the hub page (participant 6, phase 2); (3) the preference to first view a video that explains the application’s features and functionality before viewing the video that provides information about empathy (participant 5, phase 2); (4) the need to better explain that the “black marks” in the video timeline indicate tagged instances (participants 1 and 3, phase 1; and participant 6, phase 2); and (5) the frustration with not knowing how to upload and convert videos to MP4 format (participant 8, phase 3).

Overall, participants’ main comments were related to their encounters with navigational and content issues. Addressing these were a priority for the research assistants when making application refinements to enhance participants’ experiences in subsequent phases. Application refinements included, for example, creating a narrated video with screenshots of video-tagging pages as well as pop-up instructions for tagging response options on the application’s video-tagging exercise page. Screenshots of application pages were also inserted in the PDF training documents to enhance the use of the application. In addition, minor adjustments were made to the sign-in and log-out functionalities. Owing to inconsistent perceptions of the length of training portal documents, we retained the option for participants to watch the video, read the training documents, or do both. Most issues were addressed, except for those that required substantial development efforts beyond the scope and resources of the current project, including (1) attaching the stamped and tagged timeline to the video and (2) creating a graphic figure versus a CSV file to compare tags and one’s total empathic accuracy score with future scores.

### Quantitative Analysis

#### Performance Metric Results for Phases 1 and 2

Multimedia Appendix 5 displays the performance metric results for task completion, the number of errors, time on task, and the number of mouse clicks. The appendix also compares participants’ performance with standard metrics (or the “expected results” of participants based on the remote observer’s [PRB] recorded performance metrics) to facilitate interpretation and a better understanding of which tasks could be improved. Some tasks (eg, video uploading) were more controlled than others (eg, video tagging) because there was less within-task flexibility. More controlled tasks enabled more meaningful comparisons between standard metrics and participants’ performance. Notably, even though some tasks took participants longer or more clicks than the corresponding standard metrics, they still attained *task completion* for all 11 tasks. Participants made the most *errors* with “creating a new account” and “video upload.” The small sample size precluded statistical comparison, but visual analysis indicated that participants’ time on task and mouse clicks became more consistent with the corresponding standard metrics, so there may have been some improvement. Therefore, additional monitoring is required. The average number of errors across the 11 task scenarios was 1.36 errors. The average *time on tasks* suggested that “creating a tag” was the most difficult task. Of note, visually, there was improvement in “creating a tag,” which took participants only 100 seconds in phase 2 as opposed to the corresponding standard metric of 120 seconds. Consistent with our standard metrics, the tasks with the most *mouse clicks* to complete were with “create a new account” and “creating a tag.” The number of mouse clicks ranged from 1 click (exporting tags to the CSV file; standard=1 click) to 13.67 clicks (creating a new account; standard=9 clicks; note that phase 2 participants completed this task in 9 clicks, indicating improvement). Updating information, sharing the tagged video, and resetting password took slightly longer and involved more mouse clicks than the corresponding standard metrics and will require ongoing attention.

#### System Usability Scale Responses in Phase 3

With only 25% (2/8) of students participating in phase 3, only descriptive results are reported (Multimedia Appendix 6). The students’ scores were 65% (participant 8) and 90% (participant 7); scores >68 indicate a need for minor improvement in the design [40]. The strongest positive sentiments were for “I thought the IYS app was easy to use” (strongly agree to agree), “I found the IYS app unnecessarily complex” (both stated disagree), “I thought there was too much inconsistency in the IYS app” (both stated strongly disagree), and “I found the various functions in the IYS app were well integrated” (strongly agree to agree). Inconsistent responses were provided for “I would imagine that most people would learn to use the app very quickly” (disagree to agree) and “I think that I would need the support of a technical person to be able to use the app” (strongly disagree to neutral). One of the participants said that she spent 2 to 3 hours, or an average of 45 minutes, engaging with the application 3 to 4 times over the past week. Although the time spent with the application was not reported, the other participant stated that she “went through” the application once over the past week.

Upon completing the scale, one of the participants’ closing thought was, “Yes, I think’s it’s like a cool app to...It would be interesting to like...kinda do it with your friend and just see like what you do score and see if you are...like...actually on the same page as them, and I think that’s a really good idea” (participant 7, phase 3). The other participant shared, “I know this is kinda like an acting thing, but I feel like maybe if you're not in covid, we can find like a random person to talk to. I thought that would be cool because you know your friends and family” (participant 8, phase 3). Despite not having engaged with the application’s full video-capture, annotation, and feedback functions, both participants expressed interest or saw value in using them.

## Discussion

### Primary Findings and Comparison With Prior Work

This study addresses the gap in technology development where health care educators need more innovative tools to supplement classroom methods to engage students in learning empathy. The IYS application was designed to expand accessibility, ease self-directed learning, and provide compelling exercises for self-reflection and perspective taking and feedback on the student’s inferencing ability. Student participants’ feedback was addressed in application refinements before advancing to the next study phase. Input gathered in this manner signals a solid integrative knowledge translation approach, as we examined student participants’ responses to refine the application for future uptake in educational curricula.

Health sciences education has always attracted students with varying characteristics (eg, age and gender) that impact their expectations, values, norms, and learning behaviors [43]. This study’s sample consisted of men and women aged 20 to 35 years from an undergraduate nursing program located at the University of Manitoba. All student participants used a laptop daily to research information, send emails and messages, and engage with web-based education applications. Rising application use among students suggests greater acceptance of technology-based learning in nursing education [44].

Participants’ technology prowess was evident in their expectations to navigate quickly, encounter interactive web pages, and readily access resources while using the application, especially during the video annotation exercise. Several participants wanted to use the application quickly, similar to an on-demand service. However, IYS is an educational application that requires users to take time to contemplate content and perform exercises. We need to better harness students’ attention spans with more appealing content and a compelling environment where they feel committed to engaging fully with the app. Extrinsic motivators (reward points, badges, and leader boards) can foster engagement in learning [45].

Furthermore, not all participants were skilled in uploading or downloading videos as part of using the application. This was most evident in the wide variation in the System Usability Scale scores provided by the 2 phase 3 users (ie, 65% and 90%) who had expressed varied comfort levels working with technology. Because one score was lower than 68 (indicating marginal usability), additional design adjustments are warranted. The think-aloud comments provided by the low-scoring user indicated their limited computer technology skills, particularly working with videos, which made them feel hesitant to use the application. Both phase 3 users recommended the provision of instructions on how to convert a video to MP4 format. Supplemental instructions need to be provided on basic technology skills that we cannot assume are held by all students (eg, how to convert a video to MP4 format and video compression). On the application’s landing page, we should include a section called “Skills Needed to Use the App” on skills such as creating, uploading, and converting videos. To accommodate all users (with varied technology skills) and their varied contexts of use, we need to provide them with clear options. First, we can provide a standardized MP4 video of a prerecorded dialogue in the application for easy understanding of how to perform a video analysis. Second, users who wish to perform a video analysis of their own dialogue can be instructed to video record a “live” conversation that is done on the web (in light of any in-person restrictions) or in person with any dialogue partner. All users should be able to easily access the application’s full functionality for a meaningful learning experience and use it in varied contexts.

Participants valued perspective taking to better understand their clients or colleagues and expressed interest in their empathic accuracy scores. This is not surprising because this empathic process is “the subject of lifelong and continuous education” of nurses [46]. Similar sentiments were reported in the IYS pilot study with students [28,29]. However, 13% (1/8) of participants said that they will have no use for the app once they are better skilled in comprehending others. Sherf and Morrison [47] described that self-efficacy can “stand in the way” of engaging in perspective taking or seeking feedback because of the threat to one’s ego or one’s image (appearing incompetent), low perceived value in feedback, faulty assumptions about one’s inferencing abilities, or perceived effort to step outside of one’s usual cognitive routine. Their evidence also shows that when individuals were nudged to engage in perspective taking, they saw value in receiving “diagnostic information” and made corrections to their inferences about clients. Unfortunately, participants in this study did not immerse themselves as perspective takers in a “live” recorded dialogue with a partner, perform video analysis, or receive feedback because of the COVID-19 protocols, which would have brought greater meaning to using the application [6]. The experiential aspects of the application need to challenge students in their self-reliant thinking and assumptions about their empathy skills and promote appreciation for others’ viewpoints. Furthermore, the provision of empirical information on the unreliability of self-reports on empathy skills [48] and the value in seeking corrective feedback [47] could ignite curiosity and motivation to test one’s empathy skills.

Participants held strong preferences for how they wanted to learn perspective taking (reading documents, viewing videos, or both). One of the participants liked learning using a combination of technology use and in-person interaction. Clearly, different preferences for how to learn and engage with content and technology require our ongoing attention [49], as guided by the adaptive e-learning process. The adaptive e-learning process provides users with a choice in how and when they access and learn content to enhance their satisfaction with the application [50]. Student insights help (1) make the application more user centered [51], (2) increase popular uptake by ensuring fit between student contexts and the application [52], (3) provide options for users to consume educational content in different ways [53], and (4) ensure easy and convenient access to the content [54]. Students’ input has been vital in refining the application’s structured learning activities, technology features, and functionality to create a compelling learning experience that arms them with essential clinical empathy and technology skills [55].

### Limitations

Owing to limited funding and scheduling challenges, we were unable to recruit a larger sample as planned. The small sample resulted in low statistical power to test for differences in performance metric indicators between phases 1 and 2 and to more rigorously analyze System Usability Scale responses in phase 3. Owing to the COVID-19 restrictions, participants did not gain a full appreciation for the application’s main use cases: (1) to learn and practice perspective taking in a “live” recorded in-person dialogue and (2) to subsequently perform video annotation and receive feedback on their performance. This requires follow-up research with a larger sample of student participants, as determined by a power analysis. We found recruiting through posters and mailing list advertisements to be less successful than recruiting in class and providing course credit [29]. Therefore, we recommend that future research explore these alternative methods. Despite these limitations, this study’s findings were helpful for application refinements.

### Conclusions

Our study aimed to contribute to the field of web-based empathy education. Student participants’ feedback provided preliminary evidence that the theoretically based IYS application is a helpful tool that extends the empathy learning journey from the classroom to the web. However, the overall findings of this study should be considered with caution because of its small sample size. Further testing is required with a larger sample of students who will fully immerse themselves in the application’s main functionalities of providing users with the opportunity to analyze themselves in a “live” video-recorded dialogue and providing feedback on their perspective-taking performance. Before advancing to this step, we will refine the application’s educational materials, insert more instructional pop-up messages and interactive images on web pages, provide linkages to resources on how to convert and compress videos, and fix minor glitches based on student feedback. Improvements such as these make IYS and other similar applications a better educational experience.

## Appendix

Multimedia Appendix 1 Performance metric tool.

Multimedia Appendix 2 Participant characteristics.

Multimedia Appendix 3 Participant feedback on testing and action taken.

Multimedia Appendix 4 Phases 1 to 3: most liked and least liked responses.

Multimedia Appendix 5 Performance metric results for phases 1 and 2.

Multimedia Appendix 6 System Usability Scale responses for phase 3.

## Abbreviations

IYS: In Your Shoes

## Data Availability

The data sets generated and analyzed during this study are available from the corresponding author upon reasonable request.
