# Supplementary material for: Usability Testing of a Web-Based Empathy Training Portal: Mixed Methods Study
Source: JMIR Form Res. 2023 Apr 4;7:e41222. doi: 10.2196/41222 (PMC10131903; doi:10.2196/41222)
Supplement: Multimedia Appendix 1 [file formative_v7i1e41222_app1.docx]

Multimedia Appendix 1. Performance Metrics Tool

| Task | Start Time | # Mouse Clicks | Completion Time | Successful Completion | Error  Example | Error  Example | Error  Example | Overall Comments |
| --- | --- | --- | --- | --- | --- | --- | --- | --- |
| Create a New Account |  |  |  | Comments | Does not enter accepted email |  |  |  |
| Log in |  |  |  | Comments | Does not enter accepted email | Fails to enter correct password |  |  |
| Video Upload |  |  |  | Comments | Fails to Click on Correct Button/Links | Fails to Upload Video in the required format | Fails to add titles |  |
| Create a Tag |  |  |  | Comments | Fails to Click on Correct Button/Links | Fails to add comment to tagging box |  |  |
| Update Existing Tag |  |  |  | Comments |  |  |  |  |
| Share Tagged Video |  |  |  | Comments | Fails to enter acceptable email address |  |  |  |
| Export Tag to CSV format |  |  |  | Comments | Fails to find the correct link |  |  |  |
| Download a tagged video |  |  |  | Comments | Fails to find the correct link |  |  |  |
| Update information |  |  |  | Comments | Fails to enter required information in required format | Fails to enter password |  |  |
| Sign-out |  |  |  | Comments |  |  |  |  |
| Forgot password |  |  |  | Comments | Fails to enter acceptable email address | Fails to click on correct link or button | Fails at entering the email address |  |
