# Supplementary material for: Usability Testing of a Web-Based Empathy Training Portal: Mixed Methods Study
Source: JMIR Form Res. 2023 Apr 4;7:e41222. doi: 10.2196/41222 (PMC10131903; doi:10.2196/41222)
Supplement: Multimedia Appendix 2 [file formative_v7i1e41222_app2.docx]

Multimedia Appendix 2. Participant Characteristics (n = 8)

| Variable | N (%) | Mean (years); SD |
| --- | --- | --- |
| Age (years)  Range: 20 to 35 years | -- | 24 (5.07) |
| Gender  Male  Female | 2 ( 25%)  6 ( 75%) |  |
| Years in Nursing Program  3  4 | 3 ( 37.5%)  5 ( 62.5%) |  |
| Devices Used Commonly*  Laptop  Smartphone  Tablet  Computer  *total devices used by students | 8 (100%)  6 ( 75%)  3 ( 37.5%)  2 ( 25%) |  |
| Brand Names Commonly Used*  Apple  HP  Windows  Toshiba  Huawei  Sumsung  One Plus 5  *total brand names for devices used by students | 11  2  2  1  1  1  1 |  |
| Operating System*  Mac OS  Windows  Android  *total operating systems on devices used by students | 11  5  3 |  |
| Frequency of computer or laptop use  Daily  Weekly | 6 (75%)  2 (25%) |  |
| Frequency of Smartphone or Tablet Device  Daily | 3 (100%) |  |
| How above devices are used?  Research  Email  Instant Messaging  Gaming  Online Education  Chat Room  Shopping  Word processing  Spreadsheets  Databases  Pay bills  Banking  Multi-media projects  Social Media | 8 (100%)  8 (100%)  8 (100%)  3 (37.5%)  8 (100%)  6 (50%)  8 (100%)  5 (62.5%)  4 (50%)  3 (37.5%)  8 (100%)  8 (100%)  8 (100%)  8 (100%) |  |
| How often do you upload or download videos?  Never or Seldom  2 to 4 times a month | 5 (62.5%)  3 (37.5%) |  |
| How often do you use a web browser application as part of your online education training?  Seldom or Never  Daily  2 to 4 times a month | 2 (25%)  5 (62.5%)  1 (12.5%) |  |
| If you use a web browser for online training, describe platforms used  Chrome | 8 (100%) |  |
| Device used in this usability testing session  Laptop  Computer  Tablet | 6 (75%)  1 (12.5%)  1 (12.5%) |  |
| Brand name of devise used in this usability testing session  Mac  Huawei  PC (MS windows)  Toshiba | 5 (62.5%)  1 (12.5%)  1 (12.5%)  1 (12.5%) |  |
| Used wifi with this device during the usability testing session  Yes | 8 (100%) |  |
