# Supplementary material for: Usability Testing of a Web-Based Empathy Training Portal: Mixed Methods Study
Source: JMIR Form Res. 2023 Apr 4;7:e41222. doi: 10.2196/41222 (PMC10131903; doi:10.2196/41222)
Supplement: Multimedia Appendix 3 [file formative_v7i1e41222_app3.docx]

Multimedia Appendix 3. Participant Feedback on Tasks and Action Taken

| **Versions reviewed, participant perspectives** | **Feedback Area** | **Overview of feedback on 11 Key Features or Functions** | **# of Users** | **Action taken** |
| --- | --- | --- | --- | --- |
| **Phase 1** Undergraduate nursing students (n = 3) | **Function**  **Function**  **Navigation**  **Navigation**  **Navigation**  **Navigation**  **Navigation**  **Content**  **Content**  **Function**  **Function**  **Appearance**  **Content**  **Content**  **Content**  **Function**  **Function**  **Appearance** | **Create a New Account**  -Pictures not Clickable  **Log-in**  -Verification Email goes to Junk Mail  **Video Upload**  **Creating a Tag**  -Confused about the drop-down menu and the third dropdown where you were clicking self  - Unclear how to tag the video after reading the instructions  -Confused over video-tagging. Seeing a video with tags on it might help participants understand what is expected of them based on the 1st participant’s tags to make inferences on.  **Updating an Existing Tab**  **Tagged Video Sharing**  - Needed reminding to click directly on top of the target’s black marks on the timeline.  **Exporting Tags to CSV**  -Unable to identify the dialogue partners’ tags  -Expected to see video attached to tagged data  -Suggested a graphic instead of an excel spreadsheet of tags; would be nice to add to the graphic one’s ‘future’ scores to compare along with dates.  **Downloading a Tagged Video**  **Updating Information**  **Sign out of Log In Account**  -The participant had to click the sign out button twice. The first click brings them back to the home page and the second click actually signs them out.  **Forgot Password**  -The forget password section DOES let one use the same password.  **General Comments**  **Landing Page:**  -Felt the landing page font size for “anyone who wishes to better understand their clients’” is a little small.  **Training Portal:**  - Confused over video-tagging and perceiver and target tagger roles  -Some screenshots of someone actually tagging a video would be helpful  -“wordy”; “extensive”;  “overcomplicated”; “redundant” after watching the video  -Desires accessible training portal documents while engaging with app functionality later (e.g., video-tagging exercise)  -Have the PDF documents embedded into the Step dropdown menu; just click to open.  Use of more graphics to make the training portal steps more interesting. | 1  3  -  1  1  1  -  1  1  1  1  -  -  3  1  1  1  1  3  1  1  1 | Addressed, images made distinguishable, created gif screenshots  Addressed, email configuration changed to ensure that mails turn up in the inbox  -  Addressed, on the video-tagging page, we added pop-up descriptions of drop-down menu lists of response options for video-tagging instances; created an information button for how to provide a context sentence.  Addressed, above.  Addressed, created a narrated video with screenshots of video-tagging pages on how to tag instances in the video; included descriptions of drop-down menu  lists of response options.  -  Addressed, an error message appears after error committed; monitor for user complaints  Addressed; created different font colors for perceiver and target tags  Not addressed; this would involve substantial development efforts and was beyond the scope and resources of the project.  Not addressed; users can create their own CSV graphics if desired.  -  -  Not addressed; this happened because the participant clicked outside the login/signup popup which resulted in him going back to the home page; this is expected app behavior.  Not addressed, no change required. We don't think this is something that needs to be changed  Addressed, with larger font size  Addressed, in the narrated video-tagging video and in Training Portal pdf step 7 document; added pop-up explanations of tag response options  Addressed, created a narrated video with screen shots of how to tag  Not addressed, some users like having thorough pdf documents to review.  Not addressed, users can keep pdf document pages open when the open the video-tagging page  Addressed  Addressed, inserted gif animation and interactive step cards. |
| **Phase 2**  Undergraduate nursing students (n = 3) | **Content**  **Navigation**  **Function**  **Content**  **Content**  **Function**  **Content**  **Content**  **Appearance** | **Create a New Account**  **Log-in**  **Video Upload**  -Error message about required video title, make in different color.  **Creating a Tag**  -Needed reminding to click directly on the target’s black marker to avoid getting an error message  -Difficult to tag situation  when it’s a standardized video for target to tag.  **Updating an Existing Tab**  **Tagged Video Sharing**  **Exporting Tags to CSV**  -Explain CSV  -Explain why particpant might want to export the CSV file  **Downloading a Tagged Video**  **Updating Information**  **Sign out of Log In Account**  -The user had to click the sign out button twice. The first click brings them back to the home page and the second click actually signs them out.  **Forgot Password**  **General comments:**  -reading the pdf training documents a “hassle”  -Felt both the training video and pdf documents “helpful”  -Training Portal pdf documents had variable fonts. | -  -  1  3  1  -  -  1  1  -  3  -  1  1  1 | -  -  Addressed  Addressed, error message  Not addressed, this depends on the participant’s need or learning context  -  -  Addressed, narrated video-tagging video  Addressed, as above  -  Not addressed; this happened because the participant clicked outside the login/signup popup which resulted in him going back to the home page; this is expected app behavior.  -  Not addressed, other participants preferred having both the narrated video and pdf documents on training steps to review.  Not addressed |
| **Other modifications from observing participant interaction with the app’s features and functionality.** | **Appearance**  **Function**  **Navigation**  **Navigation** |  |  | In My Videos, provided clear labelling and description for: videos that are Untagged, Tagging in Process, and Tagged.  When a tagged video page is open, there is a link to the training portal document Step 8 on how to calculate your perceptual accuracy score.  Once the 1^st^ tagger completes tagging, there is a prompt for them to email a video link to the 2^nd^ tagger to tag the video.  In The Hub, we created a Messages tab for messaging to other account participants from within the app. |
