# Supplementary material for: Usability Testing of a Web-Based Empathy Training Portal: Mixed Methods Study
Source: JMIR Form Res. 2023 Apr 4;7:e41222. doi: 10.2196/41222 (PMC10131903; doi:10.2196/41222)
Supplement: Multimedia Appendix 4 [file formative_v7i1e41222_app4.docx]

**Multimedia Appendix 4. Phases 1 to 3. Think-Aloud Responses**

Upon completion of the tasks, participants provided additional feedback about the app.

**Liked Most**

The following comments capture what the participants liked most:

**Content**

**Phase 1**

- I like this Co-inventor blurp. I saw that earlier . . . ‘cause that is a good thing to learn as a nursing student. (Phase 1, Participant 3)
- I think the training steps were good. They were laid out well in the document [i.e., the training portal pdf documents] (Phase 1, Participant 1)
- The training portal step 7 document… “A nice recap of anything that was said in other documents”. (Phase 1, Participant 3)
- Training Portal Narrated Video on 8 training steps - “OK, I think this is a really, really good opening. I feel like the video is very helpful, very constructive and very informative. It was new . . . is interesting. I liked it”. (Phase 1, Participant 3)
- “And the video that the professor made was so much better than any of the text”. (Phase 1, Participant 3)

**Phase 2**

- Landing Page – “Yeah, there's . . . like . . . quite a good line or sentence”. The user is referencing the quote on cognitive empathy. (Phase 2, Participant 6)
- Explains what cognitive empathy is and what you will get out of taking this training session (Phase 2, Participant 6)

**Phase 3**

- How it explains exactly what the application is meant to do when you open it. (Phase 3, Participant 7)
- Yeah, I think the first time when I first opened it . . . um, it was very nice and I like how it told you right away, like what the app was for and like what you're supposed to learn, um or what you're supposed to get out of it when you use it. Um, I like how it had that kind of listed. . . like I think 10 things. So, I really like that. (Phase 3, Participant 7)
- “You have . . . like . . . definitions of things when you first open the app, which I think is very helpful as well”. (Phase 3, Participant 7)
- Different kinds of empathy video . . . thought it clearly explained the difference between cognitive empathy and emotional empathy. (Phase 3, Participant 7)
- Training Portal – Step 1 - So for that video I think, um, there was a video that kind of took you to YouTube where he explained empathy and I think it was like the difference between um cognitive empathy and emotional empathy. I think that was really good to have in there. In case you don't know, like the difference 'cause they kinda sound similar”. (Phase 3, Participant 7)

**Navigation**

**Phase 1**

- “Um, the making of a user account. I think that that was pretty straightforward. I like that there was the screenshot, but then when you try to click on it, it's just a picture”. (Phase 1, Participant 1)
- “I like this setup . . . it’s very intuitive”. Making reference to the Statistics page to click on the timestamped tags to open up a pop-up window that shows both perceiver and target tags. (Phase 1, Participant 3)

**Phase 2**

- Training Portal Page – “The explanations were good”. “I like the idea of having all the explanations come before all of the steps”. (Phase 2, Participant 5)
- Training Portal documents – “And I appreciate that on the last one it says go to step three at the end of the document. That was helpful. Very good for kind of tracking”. (Phase 2, Participant 5)

**Phase 3**

- “But yeah, I understood everything . . . like I didn't have any questions or I didn't feel lost at any point, and I think it's good”. (Phase 3, Participant 7)
- “I like how it clearly separates the videos in the My Videos section. You can easily tell which videos have not been tagged, which are in progress, and which are completed”. (Phase 3, Participant 7)
- “Then it was easy to navigate, easy to like . . . find everything”. (Phase 3, Participant 7)
- “But overall, the app I found it easy to like . . . navigate. Um, I didn't have problems finding like anything”. (Phase 3, Participant 7)

**Functionality**

**Phase 1**

- “I guess resetting the password was pretty quick”. (Phase 1, Participant 1)
- “I mean, I think the tagging process was pretty easy”. (Phase 1, Participant 1)
- “It's very interesting 'cause it let me see other peoples’ thoughts and perspectives” (Phase 1, Participant 2)

**Phase 2**

- “The application was pretty straightforward”. (Phase 2, Participant 4)
- “No confusion, all the tabs are again straightforward, easy to use”.  (Phase 2, Participant 4)
- Statistics page – “Yeah, that's really good. I like this page”. This user described how good it would be to actually do a live recording with a dialogue partner to see how scores align. (Phase 2, Participant 5)
- Video-tagging page – “Quite straightforward”. (Phase 2, Participant 4)
- Overall – “I think it was pretty much fine and I think everything . . . like . . . all the videos and tagging process was like . . . pretty easy to understand”. (Phase 2, Participant 5)

**Phase 3**

- “. . . when I actually did get to tagging the videos it was quite easy, easy to do . . .” (Phase 3, Participant 7)
- Overall – “Yeah, I think it's a really like cool app to use. It would be interesting to like . . . kinda do it with your friend and just see like what you do score and see if you are . . . like . . . actually on the same page as them, and I think that's a really good idea”.  (Phase 3, Participant 7)

**Appearance**

**Phase 2**

- The Hub – graphics “were quite good”. (Phase 2, Participant 5)
- The Hub, “Yeah, and I'm seeing that these icons are very helpful because they do link back to what you have here, so I think that's good. The black and white . . . “ (Phase 2, Participant 5)

**Phase 3**

- “I found the In Your Shoes web browser app visually appealing”. (Phase 3, Participant 7)

**Liked Least**

The following comments capture what the participants liked the least:

**Content**

**Phase 1**

- “The instructions for it . . . like . . . the PDF that you had me read. They were a little bit extensive but . . . like . . . I was thinking like, wow this is a lot of information for me to comprehend all at once.” (Phase 1, Participant 2)

**Phase 2**

- Landing page – Outcomes – “. . . whenever I see like a chart or you know a diagram, I might pay more attention to it so . . .”. Admits to glossing over landing page information even the Key Features information. (Phase 2, Participant 6)
- “I’d say the only thing was . . . like . . . the steps . . . reading all the different documents were kind of a hassle. I just . . . that's why I skimmed through most of it so . . .”. (Phase 2, Participant 4)

**Phase 3**

- Be more clear about having a dialogue with different partners besides family and friends. (Phase 3, Participant 8)
- Felt the learning activities were too long and preferred a mix of self-directed activities and instructor-led activities. (Phase 3, Participant 8)
- Took the user a couple of hours to watch the videos and do the reading…afterwards easy to use. (Phase 3, Participant 7)

**Navigation**

**Phase 2**

- Training Portal – “But personally I like to have . . . like . . . the explanation presented on the same page as whatever step needs to be taken. So, if it was . . . like . . . a recorded video then it would be like the explanations for how to record a video directly on the page where you do record a video. But, otherwise like it was fine, it was navigational and because the PDF documents opened in a separate page automatically . . . you can just toggle there . . .you know if you don't know what's going on”. (Phase 2, Participant 5)

**Phase 3**

- “When I opened the training portal, this statement confused me: “Based on our research, we recommend that you take the following eight steps or choose to watch the video to build perspective-taking skills”. I was confused because it said to do the following 8 steps or to watch the video. So, I watched the video, but the narrator also brought up reading the documents listed below in the 8 steps. I feel the documents went into more detail than the video”. (Phase 3, Participant 7)

**Functionality**

**Phase 1**

- Unsure of where the app would be used. (Phase 1, Participant 3)
- Felt an instructor is needed to use the app. (Phase 1, Participant 3)

**Phase 3**

- “I’m not used to converting videos to .mp4, so I found it hard to find a good website that could do so”. (Phase 3, Participant 8)
- Found uploading a .mp4 video “too techie” to do. (Phase 3, Participant 8)
- “Also, the link that’s in the portal shows you how to convert in iMovie to .mp4 but you need to already have the video downloaded on your computer and I wasn’t sure how to download it from YouTube”. (Phase 3, Participant 7)
- Difficult to use in real-time. “I think it's like good practice to kind of understand what people are . . . like thinking . . . but it would be hard to take . . . like . . . a real-life situation . . . like . . . when you're in the moment and kind of obviously put it into the app and . . . like . . . try it and see if you are understanding people”. (Phase 3, Participant 7)
- Finds video-tagging stressful because she wanted to “get it right”. (Phase 1, Participant 3)
- First time use is a bit challenging but with 2^nd^ time use it was easy to do the tagging exercise. (Phase 3, Participant 7)
- Difficult to use app and video-recording ‘real-time’ conversations. Better to use standardized videos to see how one’s perceptual accuracy is. (Phase 3, Participant 7)
- “I could just see people who aren't comfortable online having trouble, but they usually have trouble with anything to do with computers”. (Phase 3, Participant 7)

**Other Recommendations for Improvement**

**Phase 1**

**Phase 2**

- Landing Page – User suggests at the end of the Landing page to guide what to do next (e.g., Go to Training Portal). (Phase 2, Participant 5)
- User preferred to go first to The Hub and not the Training Portal. More direction is required such as putting inside The Hub instructions to first go to the Training Portal (if new to the app). (Phase 2, Participant 5)
- Video introduction to empathy. Preferred instead to first hear about the app features and functionality and not about empathy. “But so that threw me off a little bit, but otherwise, so far so good. The only thing that I want to note is there's no like introduction here . . . it was not immediately about . . . like . . . what the In Your Shoes app is, so you're kind of just thrown into like the types of empathy and I'd like to hear a little bit about . . . Like you are participating in the In Your Shoes thing. This is going to be what's going on . . . like . . . in some way, just to give us a little bit of a background 'cause she's throwing us right into how things are going to work and we're still kind of like, well, what are we doing in a way?” (Phase 2, Participant 5)
- Training Portal page - Provide instructions to first, watch the video that narrates the 8 training steps and then read the supplement pdf documents of 8 steps described in the video. (Phase 2, Participant 5)
- Training Portal Video (narrated review of 8 training steps), made reference to steps in pdf documents that accompany the video. “The only thing is that I didn't actually realize that she was referring to these steps. So maybe I missed that, but it might be useful for her to say like below are underlined the eight steps of this program so that we can kind of like read along with what she's saying so that we kind of track it a little better”. (Phase 2, Participant 5)
- Training Portal page – Introduce to users that they have an option to watch the narrated video or read the pdf training documents that described 8 training steps. (Phase 2, Participant 5)
- Training Portal Step 4 pdf document – Define who the ‘dialogue partner’ is for users. (Phase 2, Participant 5)
- Training Portal Step 4 pdf document – Energize the pages. For example, use more sub-headings to highlight key information; “Otherwise it is easy to gloss over information presented all in bullet points”. (Phase 2, Participant 5)
- Training Portal Step 5 pdf document – Tell users what behaviour or attitudes to focus on. Users need more concrete direction for the recorded dialogue. (Phase 2, Participant 5)
- Training Portal Step 7 documents – Consistency of font is required across the document. “I just took note that the formatting of this PDF is completely different from all the other ones. It's got a different font, different styles of heading and different spacing in between the different headings. This one looks better. It's cleaner, it makes more visual sense”. (Phase 2, Participant 5)
- Suggestion for step-by-step pop-ups to engage with video-tagging. (Phase 2, Participant 6)
- Video-tagging – “It just it took me a little bit of time to understand what is tagging at first until after I watched I video and then you know, did all those things. That should be fine but ... (Phase 2, Participant 6)
- Um, one thing I think it was a little bit confusing for me when I was asked to click the . . . you know, the black sign and then I could only leave comments when I click the black sign”. The user is referring to the black marker seen when tagging videos that have been shared to you. Also leaving a comment about the context or situation of the tagged instance was confusing at first to the user. (Phase 2, Participant 6)
- Video-tagging: Black markers – Suggestion is to leave a “pop up with, uh, a small window, like telling, telling the viewers why you need to click . . . black markers and then explain what you see. Explain what black markers are cause I couldn't understand what black markers are like right away. User is suggesting that a popup window explaining the black markers on the timeline mean”. (Phase 6, Participant 6)
- “I guess maybe I didn't read the instructions well enough, but like tagging other peoples’ videos where you have to click on the black mark”. (Phase 2, Participant 4)
- Editing video-tags – Insert a pop-up or brief window earlier on the tagging page about editing tags; placement at end of the page is not good notice for the user. Expand on explaining the tagging dimensions (i.e., thoughts versus feeling, tone, and situation or context). (Phase 2, Participant 5)

**Phase 3**

- Training Portal Video - Narrated video on training portal steps and video-tagging video are good but more narration is needed on other app functions; e.g., like how to upload a video is required. (Phase 3, Participant 7)
- Converting videos – Did not know how to download a YouTube video to desktop and then convert to .mp4 to upload to the app. Suggest providing a link to a video to download and then convert for the app. (Phase 3, Participant 7)
- References in the app are somewhat old and need more recent articles (Phase 3, Participant 8)
