# Supplementary material for: Usability Testing of a Web-Based Empathy Training Portal: Mixed Methods Study
Source: JMIR Form Res. 2023 Apr 4;7:e41222. doi: 10.2196/41222 (PMC10131903; doi:10.2196/41222)
Supplement: Multimedia Appendix 5 [file formative_v7i1e41222_app5.docx]

Multimedia Appendix 5. Performance Metrics Results for Phase 1 (n = 3 users) and Phase 2 (n = 3 users)

| Task # | Heat # | Task Completion | # of Errors  (mean) | Time on Task  (mean) | # of Mouse Clicks*  (mean) |
| --- | --- | --- | --- | --- | --- |
| 1 Create New Account | 1  2 | 3 users  3 users | 2  1 | 3.33 minutes  1.50 minutes | 13.67  9.00 |
| 2 Log-in | 1  2 | 3 users  3 users | 2  0 | < 0.00 minutes  < 0.00 minutes | 2.67  3.33 |
| 3 Video Upload | 1  2 | 3 users  3 users | 2  2 | 1.67 minutes  1.00 minutes | 5.33  5.00 |
| 4 Creating a Tag | 1  2 | 3 users  3 users | 0  1 | 4.67 minutes  4.00 minutes | 8.00  11.00 |
| 5 Updating Existing Tag | 1  2 | 3 users  3 users | 0  0 | 0.33 seconds  0.33 seconds | 5.00  5.00 |
| 6 Tagged Video Sharing | 1  2 | 3 users  3 users | 0  0 | 0.33 seconds  0.66 seconds | 2.67  4.00 |
| 7 Exporting Tags to CSV | 1  2 | 3 users  3 users | 0  0 | 0.66 seconds  0.33 seconds | 1.00  1.00 |
| 8 Downloading Tagged Video | 1  2 | 3 users  3 users | 0  0 | 0.33 seconds  033. seconds | 1.00  1.00 |
| 9 Updating Information | 1  2 | 3 users  3 users | 0  2 | 0.33 seconds  0.66 seconds | 4.00  5.00 |
| 10 Sign out of log in account | 1  2 | 3 users  3 users | 1  0 | < 0.00 seconds  < 0.00 seconds | 2.00  2.00 |
| 11 Forgot Password | 1  2 | 3 users  3 users | 0  2 | 0.33 seconds  1.00 minute | 5.67  9.33 |

*Independent t-tests for differences between Heat 1 and Heat 2 users across all 11 tasks were not significantly difference in the number of mouse clicks.
