# Supplementary material for: Usability Testing of a Web-Based Empathy Training Portal: Mixed Methods Study
Source: JMIR Form Res. 2023 Apr 4;7:e41222. doi: 10.2196/41222 (PMC10131903; doi:10.2196/41222)
Supplement: Multimedia Appendix 6 [file formative_v7i1e41222_app6.docx]

**Multimedia Appendix 6. System Usability Scale Responses for Phase 3 (n = 2)**

| # | System Usability Scale Questions | Participant 7 | Participant 8 |
| --- | --- | --- | --- |
| 1 | I think that I would like to use the In Your Shoes Web Browsers App frequently. | Agree | Neutral |
| 2 | I found the In Your Shoes Web Browser App unnecessarily complex. | Disagree | Disagree |
| 3 | I thought In Your Shoes Web Browser App was easy to use. | Strongly Agree | Agree |
| 4 | I think that I would need the support of a technical person to be able to use In Your Shoes Web Browser App. | Strongly Disagree | Neutral |
| 5 | I found the various functions in In Your Shoes Web Browser App were well integrated. | Strongly Agree | Agree |
| 6 | I thought there was too much inconsistency in the In Your Shoes Web Browser App. | Strongly Disagree | Strongly Disagree |
| 7 | I would imagine that most people would learn to use In Your Shoes App very quickly. | Agree | Disagree |
| 8 | I found In Your Shoes Web Browser App very cumbersome (awkward) to use. | Strongly Disagree | Strongly Disagree |
| 9 | I felt very confident using In Your Shoes Web Browser App. | Agree | Neutral |
| 10 | I needed to learn a lot of things before I could get going with In Your Shoes Web Browser App. | Strongly Disagree | Neutral |
